# Donor-derived exosomes induce specific regulatory T cells to suppress immune inflammation in the allograft heart

**DOI:** 10.1038/srep20077

**Published:** 2016-01-29

**Authors:** Jiangping Song, Jie Huang, Xiao Chen, Xiao Teng, Zhizhao Song, Yong Xing, Mangyuan Wang, Kai Chen, Zheng Wang, Pingchang Yang, Shengshou Hu

**Affiliations:** 1 State Key Laboratory of Cardiovascular Disease, Fuwai Hospital, National Center for Cardiovascular Diseases, Chinese Academy of Medical Sciences and Peking Union Medical College, 167A Beilishi Road, Xi Cheng District, Beijing, 100037, China

## Abstract

To inhibit the immune inflammation in the allografts can be beneficial to organ transplantation. This study aims to induce the donor antigen specific regulatory T cells (Treg cell) inhibit the immune inflammation in the allograft heart. In this study, peripheral exosomes were purified from the mouse serum. A heart transplantation mouse model was developed. The immune inflammation of the allograft heart was assessed by histology and flow cytometry. The results showed that the donor antigen-specific T helper (Th)2 pattern inflammation was observed in the allograft hearts; the inflammation was inhibited by immunizing the recipient mice with the donor-derived exosomes. Purified peripheral exosomes contained integrin MMP1a; the latter induced CD4^+^ T cells to express Fork head protein-3 and transforming growth factor (TGF)-β via inhibiting the Th2 transcription factor, GATA binding protein 3, in CD4^+^ T cells. Administration with the donor-derived exosomes significantly prolonged the allograft heart survival. We conclude that the donor-derived peripheral exosomes have the capacity to inhibit the immune inflammation in the allograft heart via inducing specific Treg cells, implicating that administration with the donor-derived exosomes may be beneficial to cardiac transplantation.

It is accepted that allograft transplantation is one of the effective remedies to save the life for the patients with end stage heart failure^1^. One of the major drawbacks for the clinical outcome of heart transplantation is the allograft rejection^2^. Thus, to avoid the rejection is a critical point in the long-term survival of grafts. Using immunosuppressants does reduce the incidence of rejection; however, the long-term use of immunosuppressants may result in severe side effects in patients, such as an increased incidence of infections, renal failure and malignancy^3^. Therefore, to establish the long-term specific immune tolerance against donor grafts may be the most ideal strategy to render the allografts to survive, or at least to reduce the need of immunosuppressants^4^.

One of the pathological features of the cardiac allograft rejection is the immune inflammation in the heart^5^. Chronic cardiac allograft vasculopathy results in the ischemia that eventually causes allograft failure^6^. The CD4^+^ T cell-mediated delayed-type hypersensitivity (DTH) is closely associated with the cardiac allograft rejection^7^, which can be attenuated by the generation of Treg cells^8^. However, the knowledge to generate the donor antigen specific Treg cells in recipients is quite limited currently.

The regulatory T cells (Treg cells) are one of the most important cell components in the immune tolerance system^9^. A number of substances have been reported having the potential to induce immune tolerance, such as rapamycin, can induce CD4^+^ CD25^+^ Foxp3^+^ Treg cells^10^. Rapamycin treatment may lead to an increase in the number of Treg cells by promoting the *de novo* differentiation of naive CD4^+^ T cells into Treg cells by blocking the mTOR-dependent inhibition of foxp3 transcription^11^. Integrin αvβ6 can convert the latent transforming growth factor (TGF)-β to promote the development of Treg cells^12^. The protease-activated receptor (PAR)2 is also reported playing a role in the development of Treg cells recently^13^. PAR2 is a transmembrane receptor. It can be activated by cleaving the extracellular amino terminus. A number of proteases can cleave PAR2 to activate this receptor, such as trypsin and plasmin^14^. The matrix metalloproteinases (MMP) are also a large family of proteases; some of the MMPs can be carried by exosomes^15^. Whether the MMPs are involved in the development of Treg cells has not been investigated.

Our recent study showed that the cardiovascular exosomes carried Integrin αvβ6 to promote the generation of the donor antigen specific immune tolerance^16^. Others indicate that dendritic cell-derived exosomes promote the allograft heart survival^17^. Thus, we hypothesize that the donor-derived exosomes may suppress the transplantation-induced immune inflammation in the allograft heart, and so as to enhance the allograft heart survival. In this study, we observed that the donor-derived peripheral exosomes carried MMP1a, which induced the donor antigen-specific Treg cells to attenuate the T helper (Th)2 pattern inflammation in the allograft heart, and promoted the allograft heart survival.

## Results

### Administration of donor-derived exosomes suppresses inflammation in the allograft heart

**Figure 1:**
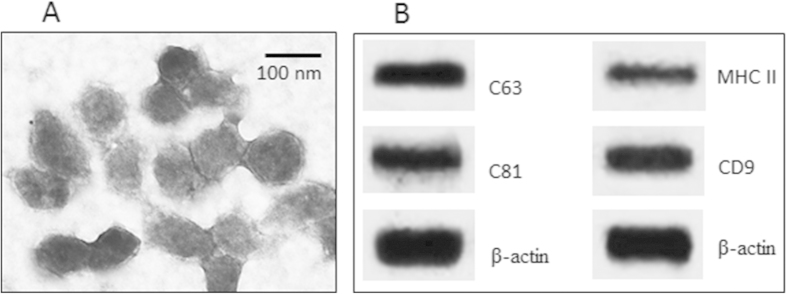
Characterization of serum-derived exosomes. Exosomes were purified from the serum. (**A**) the electron microphotography shows the exosomes. (**B**) the Western blots show the exosome markers. The data are representatives of 3 independent experiments.

The immune inflammation is a major feature of the allograft heart rejection; it is also a sign of the donor tissue tolerance has not been well established. Thus, to inhibit the inflammation may benefit the allograft heart transplantation. Our previous work indicates that exosomes contains the immune regulatory substances and the donor antigens, which can facilitate the development of the donor antigen-specific immune tolerance in the recipients^16^. Thus, we isolated exosomes from the mouse peripheral blood. As observed by electron microscopy, the exosomes were about 60–100 nm in diameter (Fig. 1A). The exosome markers, including CD9, CD63, CD81 and MHC II as suggested by the International Society for Extracellular Vesicles^18^, were also identified by Western blotting in the extracts of the exosomes (Fig. 1B).

**Figure 2:**
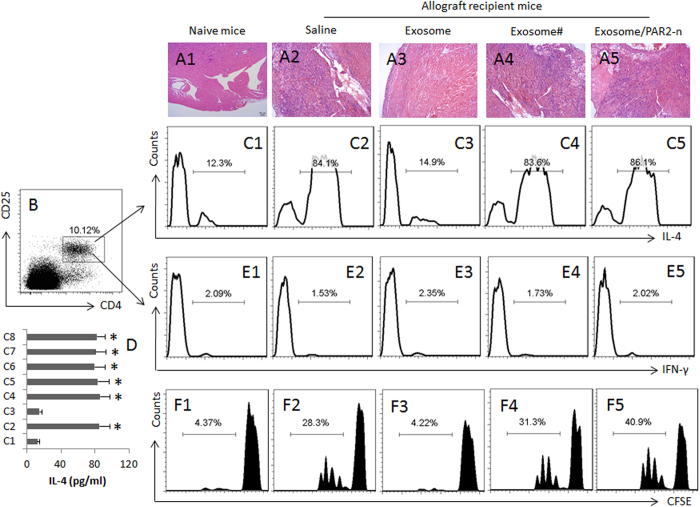
Donor-derived exosomes suppress Th2 inflammation in the allograft heart tissue. Mice were transplanted with allograft hearts. The recipient mice were treated as denoted above each column. Exosome: Mice received donor-derived exosomes. #, non-donor-derived exosomes. PAR2-n: The recipient mice are PAR2-deficient mice. (**A1-A8**) the representative images show the heart histology and the mononuclear cell infiltration (the cells were stained in dark blue) in heart tissue. (**B**) the mononuclear cells were isolated from the heart tissue and analyzed by flow cytometry. The gated dot plots indicate that the CD4^+^ CD25^+^ T cells. (**C1-C5**) the gated histograms show the frequency of IL-4^+^ CD4^+^ T cells. (**D**) the bars indicate the IL-4 levels in the culture supernatant (by ELISA; the data are presented as mean ± SD. *p < 0.01, compared to the group (**C1**) the group labels on the Y axis are the same as the histograms of (**C1-C8**). (**E1-E5**) the gated histograms show the frequency of IFN-γ^+^ CD4^+^ T cells. (**F1-F5**), the gated histograms show the frequency of proliferating CD4^+^ T cells after incubating with spleen DCs (DC:T cell = 1:5) and mouse heart extracts (1 μg/ml) for 3 days. Each group consists of 12 mice. Samples from 4 mice were pooled as one sample. The data are representatives of 3 independent experiments.

A group of mice were received the exosomes on day 0 and day 7 to generate the donor antigen-specific immune tolerance; the mice were received allograft heart transplantation on day 14, and were sacrificed one week after. The hearts were removed and processed to assess inflammation. The histology showed that a few mononuclear cells dispersed in the heart tissue of the naive mice (Fig. 2A1); in the allograft heart tissue, heavy infiltration of mononuclear cell was observed (Fig. 2A2), which was abolished in the mice pretreated with exosomes (i.p.) isolated from the heart donors (Fig. 2A3), indicating immunization with the donor-derived exosomes is capable of inhibiting the inflammation in the allograft hearts. The exosomes isolated from non-donor mice did not show the inhibitory effect on the inflammation (Fig. 2A4). It is reported that PAR2 may be involved in suppressing Th2 responses^13^; we wondered PAR2 might be also involved in the exosome-conducted inhibitory effect on the inflammation in the allograft hearts. To this end, a group of PAR2 deficient mice was received heart transplantation and treated with the donor-derived exosomes. The results showed heavy infiltration of mononuclear cells in the allograft heart (Fig. 2A5).

We then isolated the mononuclear cells from the hearts and analyzed by flow cytometry. About 10% CD4^+^ T cells were detected in the mononuclear cells (Fig. 2B). Further analysis showed that the frequency of IL-4^+^ CD4^+^ T cells was significantly increased in the allograft hearts; the increase in Th2 cells did not occur in the mice immunized with the donor-derived exosomes. The results suggest that the donor-derived exosomes can suppress the allograft-induced Th2 inflammation in the allograft hearts. To test if the suppression was antigen specific, we treated the mice with the non-donor-derived exosomes, which did not show any suppressive effects on the Th2 inflammation in the allograft hearts (Fig. 2C1, 2C4). Treating the PAR2-deficient mice with the donor-derived exosomes did not suppress the Th2 inflammation in the allograft hearts (Fig. 2C5). The results suggest a Th2 dominant status was developed in the allograft hearts. To enforce the results, we measured the levels of IL-4 in the culture. The results showed high levels of IL-4 were detected in the culture supernatant, which were in parallel to the Th2 cell number (Fig. 2D).

It is suggested that Th1 pattern-inflammation plays a role in the allograft heart rejection^19^. Thus, we checked the frequency of Th1 cells in the heart-isolated mononuclear cells by flow cytometry. The results showed that the Th1 cell number did not increase in the allograft hearts in wild mice or PAR2-deficient mice. Treatment with exosomes also did not increase the frequency of Th1 cells in the allograft hearts (Fig. 2E1-E5). The Th1 cytokine IFN-γ and cytotoxic CD8^+^ T cell cytokines, granzyme B and perforin were below the detectable levels in the culture supernatant (not shown). We also assessed the frequency of CD8^+^ T cells in the heart by flow cytometry. The results showed that CD8^+^ T cells were detected in the heart of the naive mice, which were not significantly altered by the heart transplantation (Fig. S1 in supplemental materials).

### Donor-specific Th2 cells are detected in the allograft hearts

The data reported above indicate a Th2 dominant status in the allograft hearts. To elucidate whether the Th2 cells were antigen specific, we purified CD4^+^ T cells by MACS from the mononuclear cells isolated from the allograft hearts and analyzed by CFSE-dilution assay. The results showed a donor antigen-specific Th2 cell population was detected in the CD4^+^ T cells (Fig. 2F1-F2). Immunization with the donor-derived exosomes (Fig. 2F3), but not the non-donor-derived exosomes (Fig. 2F4), inhibited the development of the antigen-specific Th2 cells.

### Donor antigen specific Treg cells are generated in recipient mice by the immunization with the exosomes

**Figure 3:**
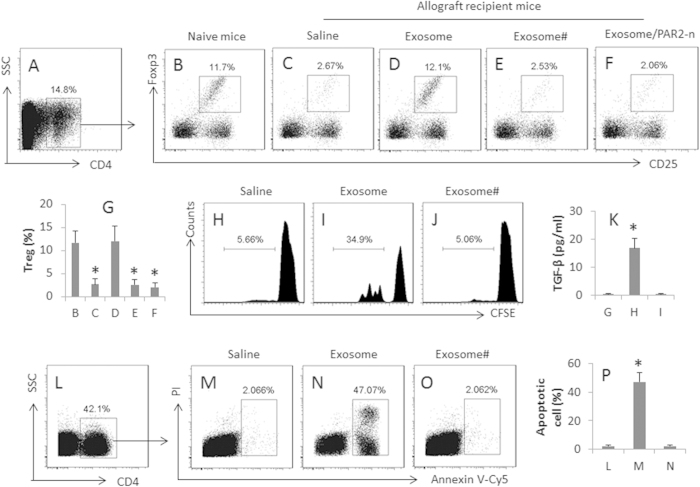
Donor-derived exosomes induce donor antigen specific Tregs. CD4^+^ T cells were isolated by MACS from the wild hearts and allograft hearts and analyzed by flow cytometry. (**A**) the CD4^+^ T cells were gated. (**B–F**) the gated dot plots indicate the frequency of Tregs in the gated CD4^+^ T cells of (**A**). PAR2-n: PAR2-deficient mice. (**G**) the bars indicate the summarized data of (**B–F**). (**H–J)**, Tregs (CD4^+^ CD25^+^ CD127^-^) were isolated from the heart-isolated T cells, labeled with CFSE, and cultured in the presence of exosomes and DCs (T cell:DC  =  5:1) for 3 days. The gated histograms indicate the frequency of the proliferating Tregs. (**K**) samples were collected from the culture medium of (**G–I)** and analyzed by ELISA. The bars indicate the TGF-β levels in the medium. (**L–O**) Tregs were isolated from the allograft hearts (the recipient mice were treated with donor-derived exosomes) and cultured with polarized Th2 cells at a ratio of 1:1 in the presence of DCs and exosomes for 48 h. The cells were stained with Annexin V reagents and propidium iodide (PI), and analyzed by flow cytometry. (**L**) the CD4^+^ T cells were gated first. (**M–O**) the gated dot plots indicate the frequency of apoptotic cells. (**P**) the bars indicate the summarized data of M-O. #, the exosomes were obtained from non-donor mice. The data of bars are presented as mean ± SD. *p < 0.01, compared to group B (**G**), or group G (**K**), or group L (**P**). Each group consists of 10 mice. Samples from individual mice were processed separately.

Treg cells and regulatory B cells (Breg cell) have the immune suppressor function, and can inhibit immune inflammation^20^. Our previous studies show that the cardiovascular endothelium-derived exosomes can induce regulatory B cells^16^. We inferred that the effect of the exosome immunization on the inhibition of the inflammation in the allograft hearts might be rendered by the generation of Breg or Treg cells in the heart. We then observed the effect of the exosomes on the generation of Breg and Treg cells in the allograft hearts. The results showed that both Treg and Breg cells were detected in the normal heart tissue. The frequency of Treg cells was much less in the allograft hearts (Fig. 3A–C,G), which was returned to the normal levels in the allograft hearts of mice received the donor-derived exosome immunization (Fig. 3D,G). Treating with exosomes from the non-donor mice did not increase Tregs in the allograft hearts (Fig. 3E–G). The frequency of Breg cells was not much different between groups (data not shown). To elucidate if the Treg cells in the allograft hearts were the donor antigen specific, we isolated CD4^+^ T cells from the hearts and assessed by flow cytometry. The results showed that more than 30% Treg cells was the donor antigen specific as showing proliferation and releasing TGF-β into the culture medium (Fig. 3H–K). The Treg cells isolated from the exosome-treated allograft hearts showed the immune suppressor effect on inducing polarized Th2 cell apoptosis (Fig. 3L–P). The results suggest that the donor-derived exosomes induce antigen specific Treg cells in the allograft hearts.

### The exosomes convert Th2 cells to Treg cells

**Figure 4:**
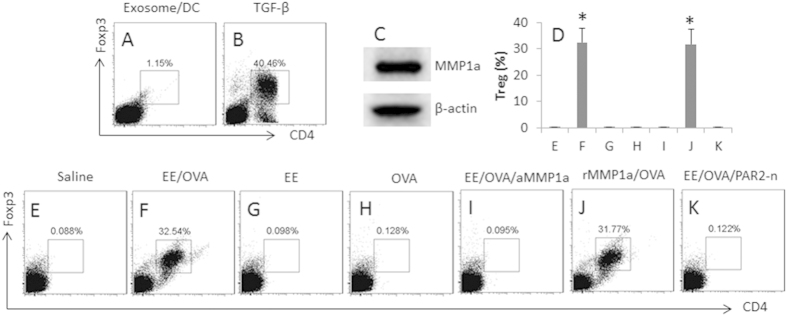
Exosome-carrying MMP1a facilitates Treg generation. (**A,B**), naive CD4^+^ T cells and DCs were isolated from the naive mouse spleen, and cultured in the presence of the exosomes and DCs, or TGF-β (20 ng/ml; using as a positive control), for 6 days. The dot plots show the frequency of CD4^+^ Foxp3^+^ Tregs. (**C**) the Western blots show the MMP1a levels in the exosome extracts. (**D**) OVA-specific polarized Th2 cells were prepared, and treated in the culture as denoted above each subpanel. The cells are analyzed by flow cytometry. The bars indicate the frequency of CD4^+^ Foxp3^+^ Tregs; the individual dot plots are presented in (**E–K**). Concentrations of the agents: EE = 2 μg/ml; OVA = 5 μg/ml; anti-MMP1a (aMMP1a) = 1 μg/ml. rMMP1a = 100 ng/ml. Ratio of T cell:DC = 5:1. The data of bars are presented as mean ± SD. *p < 0.01, compared to group (**E**). The data are representatives of 3 independent experiments.

The results of Fig. 3 imply that the donor-derived exosomes induce Treg cells in the hearts; the Treg cells suppress the Th2 inflammation in the allograft hearts. To test the inference, we isolated naive CD4^+^ T cells and DCs from the naive mouse spleen to be cultured in the presence of the exosomes for 6 days. However, no detectable evidence showed that the exosomes could directly induce Treg cells (Fig. 4A) in contrast to the positive control group (Fig. 4B). Prompted by our previous studies^13^, in which antigen specific Th2 cells can be converted to Treg cells, we postulated that the increased Treg cells in Fig. 3 might be converted from Th2 cells. To test this, we generated polarized OVA-specific Th2 cells; the cells were cultured in the presence of the extracts of the exosomes (EE) or/and OVA and DCs for 6 days. As assessed by flow cytometry, the exposure to the EE and OVA converted the Th2 cells to Treg cells (Fig. 4D–F), but not in those Th2 cells treated with either EE (Figs 4D and 3G) or OVA (Figs 4D and 3H) alone.

**Figure 5:**
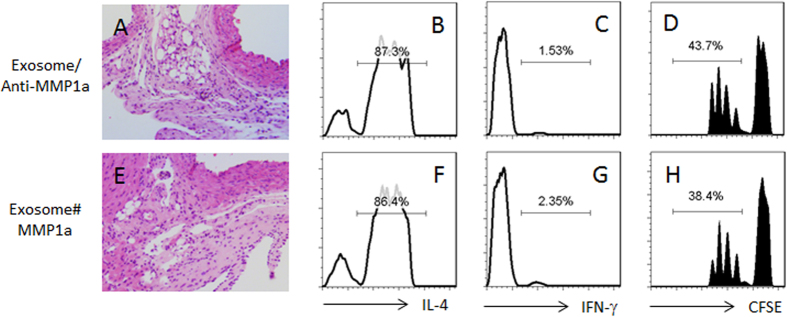
MMP1a plays a critical role in the donor-derived exosome-induced allograft tolerance. Similar to Fig. 2, the recipient mice of heart allograft were treated with the exosomes (or non-donor-derived exosomes; #) and anti-MMP1a (0.25 mg/mouse) or MMP1a (0.1 mg/mouse) on the day of surgery. (**A**) the heart histology image shows infiltration of inflammatory cells. (**B–D**) the histograms show the frequency of IL-4^+^ T cells (**B**), IFN-γ^+^ T cells and proliferating CD4^+^ T cells (**D**). Each group consists of 12 mice. Samples from 4 mice were pooled as one sample.

Because Th2 cells express PAR2^13^, the activators of PAR2 might be carried by the exosomes in the experiments of Fig. 3. Since MMP1a can activate PAR2^21^, we assessed the MMP1a levels in the exosomes. Indeed, high levels of MMP1a were detected in the exosomes (Fig. 4C). The results suggest that the exosome carrying MMP1a activates Th2 cells and to convert the Th2 cells to Tregs. To test the inference, we executed an experiment with the MMP1a neutralized by an antibody against MMP1a; the conversion of Treg cells was abolished (Fig. 4D,I). To strengthen the results, we added recombinant MMP1a and OVA to the culture, which also converted Th2 cells to Treg cells (Fig. 4D,J). On the other hand, to confirm the role of PAR2 in the Treg conversion, we treated the OVA-specific PAR2-deficient Th2 cells with the EE and OVA, which did not show any detectable Treg conversion (Fig. 4D,K). The results suggest that the peripheral exosomes can convert Th2 cells to Treg cells, in which MMP1a and PAR2 play a critical role, and the activation of antigen specific T cell receptor (TCR) is also required. To further enforce the results about the role of PAR2, with similar procedures of Fig. 3, we created an allograft heart transplantation model with PAR2-deficient mice; the mice were treated with the donor-derived exosomes; the conversion of Treg cells was not induced (Fig. 3F,G). In addition, administration of anti-MM1a Ab into allografted mice with donor-derived exosomes could abolish the inhibitory effect of the donor-derived exosomes on the inflammation in the allograft heart (Fig. 5A–D). Administration of MM1a into allografted mice with non-donor-derived exosomes could induce the immune inflammation in the allograft heart (Fig. 5E–H).

### Activation of PAR2 inhibits antigen specific TCR activation-induced GATA3 expression in Th2 cells

**Figure 6:**
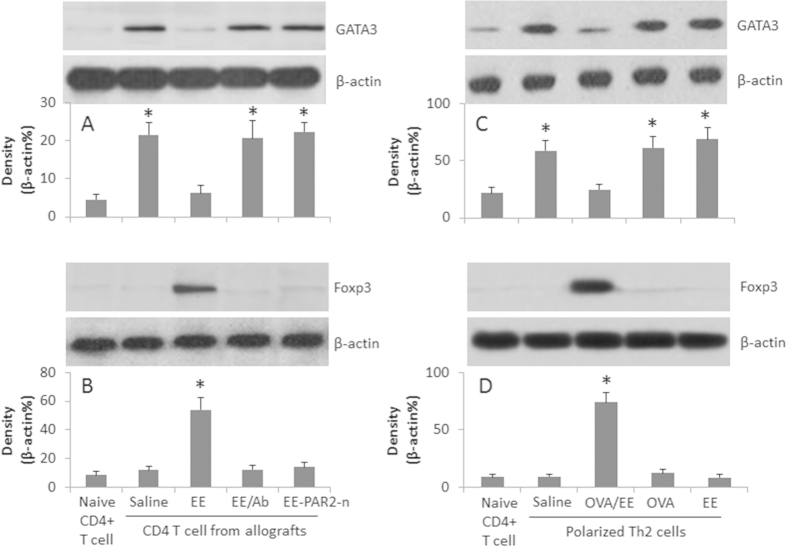
Peripheral exosomes modulate the expression of GATA3 and Foxp3 in Th2 cells. CD4^+^ T cells were isolated from the naive mouse hearts and the allograft hearts (**A,B**); OVA-specific polarized Th2 cells were prepared (**C,D**). The cells were treated as denoted below the bars. The Western blots show the protein levels of GATA3 and Foxp3. The bars below the blots indicate the integrated density of the blots. EE: Exosome extracts (5 μg/ml). Ab: Anti-MMP1a antibody (1 μg/ml). PAR2-n: PAR2-deficient mice. The data are representative of 3 independent experiments.

Fig. 2 shows that the Th2-pattern inflammation is induced in the allograft hearts. Since GATA3 is a critical factor in the Th2 polarization, the results implicate that the exposure to the exosomes and the specific antigens modulates the expression of GATA3 somehow. To test the inference, we assessed the levels of GATA3 and Foxp3 in the isolated CD4^+^ T cells from the allograft hearts. The results showed higher levels of GATA3 and low levels of Foxp3 in the cells from allograft hearts (Fig. 6A,B). Treatment with exosomes markedly down regulated the levels of GATA3 and increased the levels of Foxp3 and TGF-β (Fig. 6A,B). To strengthen the results, we executed *in vitro* experiments to assess the expression of GATA3 in the polarized Th2 cells after exposure to EE or/and OVA in the presence of DCs. The CD4^+^ T cells were isolated by MACS; the cell extracts were analyzed by Western blotting. The results showed that the exposure to OVA alone markedly increased the GATA3 levels in the Th2 cells while the levels of GATA3 in the Th2 cells exposed to both EE and OVA showed much lower levels of GATA3, instead, the cells showed high levels of Foxp3, which did not occur in those exposed to OVA or EE alone (Fig. 6C,D). The results suggest that the PAR2 plays a critical role in mediating the effect of the exosomes on the conversion of Treg cells from Th2 cells.

### HDAC1 and PAR2 are involved in the Treg conversion from Th2 cells

**Figure 7:**
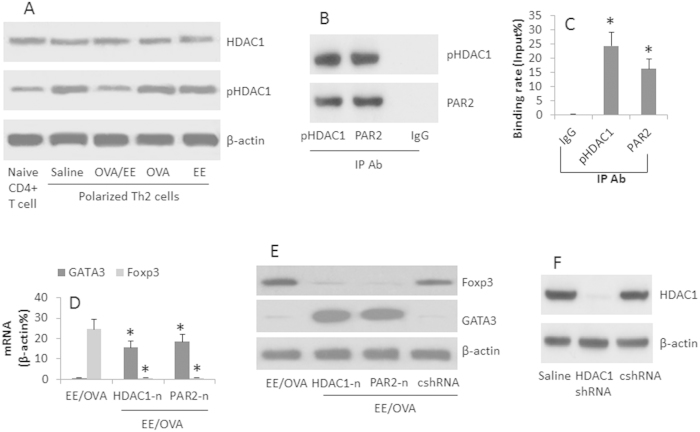
HDAC1 mediates the exosome-induced Foxp3 expression in Th2 cells. OVA-specific polarized Th2 cells were prepared. The treatment was denoted in the individual panels. The cell extracts were prepared and analyzed as indicated. (**A**) the Western blots indicate the protein levels of HDAC1 and pHDAC1 in the Th2 cells. (**B**) the Western blots indicate a complex of HDAC1 and PAR2 was detected in the cell extracts of Th2 cells (by IP). (**C**) the bars indicate the GATA3 promoter binding rate by the pHDAC1/PAR2 complexes (by ChIP). (**D**) the bars indicate the mRNA levels of GATA3 and Foxp3 in Th2 cell extracts (by RT-qPCR). (**E**) the Western blots indicate the protein levels of Foxp3 and GATA3 in the cell extracts of Th2 cells. (**F**) the Western blots indicate the results of HDAC1 gene silence. HDAC1-n: HDAC1-deficiency. PAR2-n: PAR2-deficiency. cshRNA: Control shRNA. The data of bars are presented as mean ± SD. *p < 0.01, compared to the group IgG (**C**), or the group EE/OVA (**D**). The data are representative of 3 independent experiments.

**Figure 8:**
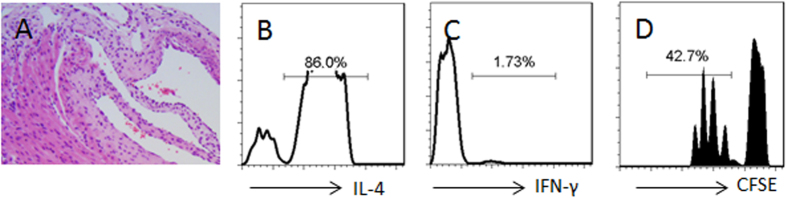
Butyrate prevents the effect of exosome on inducing allograft tolerance. Similar to Fig. 2, the recipient mice of heart allograft were treated with the exosomes and butyrate (1.2 g/kg; ip) on the day of surgery. (**A**) the heart histology image shows infiltration of inflammatory cells. (**B–D**) the histograms show the frequency of IL-4^+^ T cells (**B**), IFN-γ^+^ T cells and proliferating CD4^+^ T cells (**D**). Each group consists of 12 mice. Samples from 4 mice were pooled as one sample. The data are representatives of 3 independent experiments.

Previous reports indicate that HDAC1 activation is involved in the inhibition of GATA3^22^. We wondered if HDAC1 was also involved in the PAR2-mediated GATA3 suppression in Th2 cells as shown by Fig. 6. To this end, we assessed the HDAC1 phosphorylation in OVA-specific Th2 cells. The results showed that the levels of pHDAC1 were increased after exposure to the EE and OVA, but not in either EE alone or OVA alone (Fig. 7A). We also detected that PAR2 formed a complex with pHDAC1 in the Th2 cells (Fig. 7B). The complex of PAR2/pHDAC1 bound to the promoter of GATA3 (Fig. 7C), which decreased the expression of GATA3 and increased the expression of Foxp3 (Fig. 7D,E), which was abolished in the HDAC1-deficient (Fig. 7F) or PAR2 deficient cells (Fig. 7D,E). It is noteworthy that such a phenomenon only occurred in those Th2 cells exposed to both EE and OVA, and did not occur when the Th2 cells were exposed to EE and BSA. To enforce the results, we added butyrate sodium, an inhibitor of HDAC1, to the culture; it abolished the conversion of Treg cells from Th2 cells as well as abolished the inhibitory effect of the donor-derived exosomes on the inflammation of the allograft hearts (Fig. 8).

### Administration with donor-derived exosomes prolongs allograft heart survival

**Figure 9:**
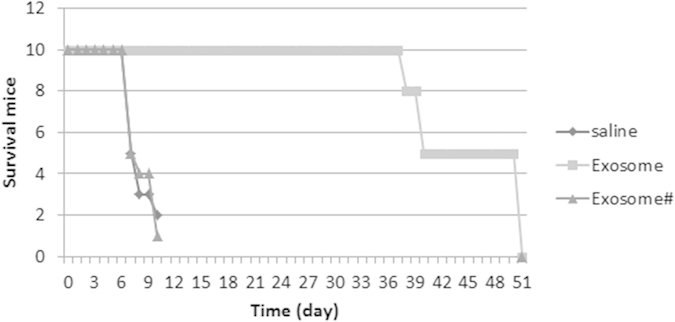
Administration with exosomes extends allograft heart survival. Mice were treated with the donor-derived exosomes, or non-donor-derived exosomes (#), or saline, and received heart transplantation. The curves show the survived mice at different time points.

Finally, we observed the effect of the donor-derived exosome on allograft heart survival. BALB/c mice were immunized with donor-derived exosomes, or non-donor-derived exosomes, or saline, and received heart transplantation. As shown by Fig. 9, ten mice treated with saline died on day 7, 8 and 10 respectively. In contrast, the ten mice treated with the donor-derived exosomes died on day 38, 40 and 50 respectively, while the mice received non-donor-derived exosomes showed similar results to those treated with saline. The results demonstrate that immunization can significantly (p < 0.01) promotes the allograft heart survival.

## Discussion

The present data show that administration with donor-derived exosomes significantly prolonged the allograft heart survival. The allograft hearts are a kind of foreign antigen. When an allograft heart is transplanted into a recipient, an adaptive immune response against the allografts can be generally induced in the host. According to the classic immunological knowledge, the induced immune response should be the Th2 pattern. The present data show that, after the heart transplantation in mice, profound mononuclear cell infiltration was observed in the allograft hearts, indicating an inflammatory status in the allograft heart tissue. Toscano *et al* also indicate that the giant cell myocarditis can be induced in the allograft hearts^23^. Similar inflammatory condition in the allograft hearts is reported in human subjects after heart transplantation^24^. Thus, it is necessary to understand the immune type of the inflammation in the allograft hearts. The present data show that, the infiltrated mononuclear cells in the allograft heart tissue are mainly Th2 cells. The fact indicates that a Th2 pattern inflammation has been induced in the allograft hearts. It is noteworthy that we found that the Th2 cells isolated from the allograft tissue were donor antigen-specific. Upon exposure to the donor antigen in the culture, the cells proliferated and released IL-4 to the culture medium. Different inflammation pattern in the mouse heart transplantation was reported. Wood *et al* found Th1 dominated inflammation in the allograft hearts^19^. The difference between us may be because the procedures are different. They transplanted hearts from BALB/c mice to C57BL/6 mice; we did in a reverse way. BALB/c mice are prone to inducing Th2 inflammation.

It is accepted that Treg cells have an immune suppressor function on other effector T cell activities so as to inhibit immune inflammation^25^. Thus, investigators have tried to generate Treg cells in the allograft recipients, or adoptively transferred the *in vitro* generated Treg cells to the allograft recipients, to promote the allograft survival^26^. Our data are principally in line with previous studies. In our study, the immunization with the donor-derived exosomes induced a plenty of Treg cells in the recipient mice. The novel aspect of our study is that the induced Treg cells are the donor antigen specific. Those Treg cells can be activated only in exposing to the specific antigens. The significance of the results is the allograft can keep these Treg cells active, so as to inhibit other inflammatory immune cell activities. The inference is supported by the histology results. The inflammation in the allograft heart tissue was inhibited in the mice immunized with the donor-derived exosomes.

The data show that the donor-derived exosomes have the ability to induce the donor antigen specific Treg cells. The functional components in the exosomes include the exosomes and the contents carried by the exosomes. Since exosomes are the plasma membrane of cells in nature, the exosome extracts can serve as the donor antigens. The contents carried by the exosomes are responsible for the generation of Treg cells in the present study. We found that the exosomes carried MMP1a, which is responsible for the generation of Treg cells. The results are supported by the addition of recombinant MMP1a to the T cell culture also induced Treg cells. Others also observed that MMPs were a generator of Treg cells^27^.

T cells are not the absorptive cells. Thus, the MMP1a must act on the surface structure to regulate the activities of the T cells. Since T cells express PAR2^13^, the MMP1a cleaves PAR2 to activate the T cells. The results showed that the MMP1a indeed cleaved PAR2 to induce naive CD4^+^ T cells to differentiate into Treg cells. Supportive data have been published by Li *et al*^21^, in which Li *et al* found that MMP1a cleaved PAR2 to induce expression of monocyte chemoattractant protein-1 by activation of PAR2 in A549 human lung adenocarcinoma cells. Our study showed that the activated PAR2 bound the pHDAC1 to inhibit the gene transcription of GATA3 in polarized Th2 cells. GATA3 is the key transcription factor of Th2 cells; it binds to Foxp3 to inhibit Foxp3 gene transcription^28^. Our data are in line with this report by showing that the inhibition of GATA3 by the PAR2/pHDAC1 complex indeed up regulated the gene transcription of Foxp3 in the cells, thus, to induce the polarized Th2 cells to differentiate into Treg cells, which show the inhibitory effects on suppression of polarized Th2 cells.

In summary, the present data show that immunization with the donor-derived exosomes inhibits the Th2 pattern inflammation in the allograft hearts and extended the allograft heart survival in mice by facilitating the generation of donor antigen specific Treg cells. The data suggest that the donor-derived peripheral exosomes have the potential to be used to inhibit the immune inflammation in the heart transplantation.

## Materials and Methods

### Reagents

The fluorochrome-labeled antibodies of IL-4, CD25, CD4, TGF-β and Foxp3 for flow cytometry were purchased from BD Biosciences (Beijing, China). The neutralizing antibody of MMP1a, ELISA kits of IL-4, IFN-γ, granzyme B, perforin and TGF-β were purchased from R&D Systems (Beijing, China). The Annexin V-Cy5 reagent kit, Imprint® Chromatin Immunoprecipitation (ChIP) Kit and protein G were purchased from Sigma Aldrich (Beijing, China). The antibodies of MMP1a, GATA3, Foxp3, HDAC1, pHDAC1, PAR2, CD9, CD63, CD81, MHC II and shRNA kit of HDAC1 were purchased from Santa Cruz Biotech (Beijing, China). The recombinant MMP1a was synthesized by GeneScript (Nanjing, China). The immune cell isolation kits were purchased from Miltenyi Biotech (Beijing, China).

### Mice

Male BALB/c and C57BL/6 mice (10–12 week old) were purchased from the Beijing Laboratory Animal Center. The DO11.10 OVA-TCR transgenic mice were purchased from the Xian Experimental Animal Center. The PAR2-deficient mice were provided by the Shenzhen Huada Gene Co. Ltd. The mice were maintained in a pathogen-free environment. The using mouse in the present study was approved by the Animal Ethic Committee at Beijing Fuwai Cardiovascular Disease Hospital. The experiments were performed in accordance with the approved guidelines.

### Isolation of peripheral exosomes

Mice were sacrificed by truncating the head; the blood was collected to isolate the serum. The exosomes in the serum were isolated by the procedures we reported previously^16^. Briefly, the serum was centrifuged at 1000 g and 8,000 g to eliminate cell debris, and subsequently centrifuged at 60,000 g. The pellet was then washed with PBS and pelleted again at 100,000 g. Isolated exosomes were resuspended in PBS and filtered twice through 0.22-μm filters. The contents of MMP1 and exosome markers, including CD9, CD63, CD81 and MHC II, in the exosomes were determined by Western blotting. The isolated exosomes were quantified by Bio Rad protein assay and visualized by electron microscopy^16^.

### Mouse heart transplantation and additional treatment

The hearts from C57BL/6 mice were transplanted into BALB/c mice, which were carried out following the published procedures^29^. Briefly, under general anesthesia, the donor heart was removed under a sterile environment. An end to side anastomosis of the donor aorta to the recipient aorta and end to side anastomosis of the donor pulmonary artery to the recipient IVC were completed using 10–0 nylon suture.

Ninety-six mice were divided into 8 groups with 12 mice per group. One group mice was used as the naive control; the rest 7 group mice were received the heart transplantation in addition to receiving saline (ip), or donor-derived exosomes (10 μg/mouse), or non-donor-derived exosomes (10 μg/mouse), or donor-derived exosomes (PAR2-deficient mice), or donor-derived exosomes and anti-MMP1a Ab, or non-donor-derived exosomes and recombinant MMP1a, or exosomes and butyrate (1.2 g/kg, ip). The exosomes were administered on day 0 and day 7; the heart transplantation surgery was performed on day 14. The anti-MMP1a Ab, or MMP1a, or butyrate was administered on day 0 and day 3 after the surgery.

### Heart histology

The mice were sacrificed by cervical dislocation on day 7 after the heart transplantation. The hearts were excised and fixed in 10% formalin for 24 h, and processed for paraffin sections and H&E staining. The heart histology was observed under a light microscope.

### Isolation of mononuclear cells from the hearts

After sacrifice, the heart was removed. In order to remove the immune cells from the blood vessels, the heart was flushed with warm (37 °C) saline (10 ml) via the venae cava inferior intubation at 20 cmH_2_O. The hearts were then cut into small pieces (2 × 2 × 2 mm) and incubated in RPMI1640 medium containing collagenase IV (0.5 mg/ml) for 2 h at 37 °C with mild agitation. The cells were filtered through a cell strainer (70 μm) and collected by centrifugation.

### Cell culture

The cells were cultured in RPMI1640 medium complemented with 10% fetal bovine serum, 100 U/ml penicillin, 0.1 mg/ml streptomycin and 2 mM L-glutamine. The cell viability was assessed by Trypan blue exclusion assay.

### Flow cytometry

The mononuclear cells isolated from allografts were fixed with 2% paraformaldehyde containing 0.1% Triton X-100 for 2 h. After washing with PBS, the cells were incubated with the fluorochrome-labeled antibodies (or isotype IgG using as controls) for 30 min at room temperature. The cells were analyzed by a flow cytometer (FACSCanto II, BD Bioscience). The data were analyzed with the software FlowJo. The data from isotype IgG staining were used as a gating reference.

### Assessment of T cell proliferation

DCs were isolated from the mouse spleen; CD4^+^ T cells were purified from the isolated allograft mononuclear cells by magnetic cell sorting (MACS) and labeled with carboxyfluorescein succinimidyl ester (CFSE; 0.5 μM). The T cells and DCs were cultured at a ratio of 5:1 in the presence of the exosome extracts (EE; 1 μg/ml), for 3 days. The cells were collected and analyzed by flow cytometry, the CFSE-dilution assay.

### Enzyme-linked immunosorbent assay (ELISA)

The levels of IL-4 and TGF-β in the culture medium were determined by ELISA with commercial reagent kits following the manufacturer’s instructions.

### Assessment of apoptotic cells

The frequency of apoptotic cells was analyzed by flow cytometry with an Annexin V-Cy5 reagent kit and stained with propidium iodide (PI) following the manufacturer’s instructions. The Annexin V^+^ or PI^+^ Annexin V^+^ cells were regarded as apoptotic cells.

### Immunoprecipitation

For detection of the complexes of PAR2 and histone deacetylase-1 (HDAC1) in the CD4^+^ T cells, the cells were lysed in lysis buffer. The lysates were precleared for 30 min at 4 °C by adding IgG1 and protein-A sepharose beads with 5% BSA. The beads were then incubated with either IgG1 (using as a control) or anti-PAR2, or anti-HDAC1, and incubated overnight at 4 °C. After washing 3 times with 1 × lysis buffer, the beads were incubated with 1 × SDS loading buffer, boiled for 10 min and subjected to SDS–PAGE.

### Western blot analysis

The total proteins were extracted from cells, fractioned by SDS-PAGE and transferred onto a PVDF membrane. The membrane was then blotted with antibodies of interest. The blots were developed with ECL. The results were photographed with a KODAK Image Station 4000 Pro (KODAK, Shanghai, China).

### Chromatin immunoprecipitation (ChIP)

To perform ChIP, the imprint ChIP kit was used. After the indicated treatment, CD4^+^ T cells were fixed wth 1% formaldehyde and digested with micrococcal nuclease to induce optimal genomic DNA fragmentation. The cells were then lysed in lysis buffer. After sonication, immunoprecipitation was performed with the samples with anti-PAR2, or anti-pHDAC1, or IgG. The non-antibody treated chromatin was set aside and used as the input DNA. Protein A sepharose beads were added to precipitate the antibody-protein-DNA complex. Then, the complex was reversibly cross-linked using proteinase K at 65 °C to obtain the IP DNA, which was purified afterward. With the IP DNA, relative abundance of DNA sequence from the GATA3 promoter region was analyzed by qPCR in a qPCR device (LightCycler 480 qPCR instrument; Roche Diagnostics Corporation). The primer sequences used are: Forward, aacctcttaagttgcgtcgc; reverse, caaggagcgtagaggaggag. The enriched DNA after qPCR was represented as % input.

### Real time RT-PCR (RT-qPCR)

The total RNA was extracted from the isolated cells; the cDNA was synthesized with the RNA with a reverse transcription kit. The qPCR was carried out in a qPCR device (LightCycler 480 qPCR instrument; Roche Diagnostics Corporation) with the SYBR Green Master Mix. Data were calculated with the method of 2^−ΔΔCt^. The primers used in this study include: Foxp3, forward, cctccactccacctaaagca; reverse, ccttgttttgcgctgagagt. GATA3, forward, tctccaagtgtgcgaagagt, reverse, tccggattcagtggttggaa.

### Th2 cell polarization *in vitro*

CD4^+^ CD25^-^ T cells were isolated from the DO11.10 mouse spleen by MACS. The cells were cultured in RPMI1640 medium in the presence of IL-4 (40 ng/ml), anti-IL-12 antibody (1 μg/ml) and anti-IFN-γ antibody (1 μg/ml) for 3 days. The cells were washed with fresh medium and cultured in fresh medium in the presence of IL-2 (10 ng/ml) for 3 more days. The rate of IL-4^+^ CD4^+^ T cells was 93.3% as checked by flow cytometry on day 6.

### Treg generation by exosome extracts *in vitro*

CD4^+^ CD25^-^ T cells were isolated from the mouse spleen by MACS. The cells were cultured in RPMI1640 medium in the presence of the extracts (EE) of purified exosomes at 2 μg/ml. The medium and EE were changed on day 3 and day 6 respectively. Sample cells were collected on day 9 and analyzed by flow cytometry. The results showed that the Foxp3^+^ T cells were 92.2%.

### Statistics

The data are presented as mean ± SD. Differences between two groups were determined by Student t test, or by ANOVA if more than two groups. A p < 0.05 was set as a significant criterion.

## Additional Information

**How to cite this article**: Song, J. *et al.* Donor-derived exosomes induce specific regulatory T cells to suppress immune inflammation in the allograft heart. *Sci. Rep.* **6**, 20077; doi: 10.1038/srep20077 (2016).

## Supplementary Material

Supplementary Information
