# Erratum: Donor-derived exosomes induce specific regulatory T cells to suppress immune inflammation in the allograft heart

**DOI:** 10.1038/srep46389

**Published:** 2017-06-30

**Authors:** Jiangping Song, Jie Huang, Xiao Chen, Xiao Teng, Zhizhao Song, Yong Xing, Mangyuan Wang, Kai Chen, Zheng Wang, Pingchang Yang, Shengshou Hu

Scientific Reports 6: Article number: 20077; 10.1038/srep20077 published online: 01 29 2016; updated: 06 30 2017.

The original HTML version of this Article listed an incorrect volume number. This has now been corrected in the HTML version; the PDF version was correct at the time of publication.

