# Author Correction: Donor-derived exosomes induce specific regulatory T cells to suppress immune inflammation in the allograft heart

**DOI:** 10.1038/s41598-021-88549-1

**Published:** 2021-04-21

**Authors:** Jiangping Song, Jie Huang, Xiao Chen, Xiao Teng, Zhizhao Song, Yong Xing, Mangyuan Wang, Kai Chen, Zheng Wang, Pingchang Yang, Shengshou Hu

**Affiliations:** State Key Laboratory of Cardiovascular Disease, Fuwai Hospital, National Center for Cardiovascular Diseases, Chinese Academy of Medical Sciences and Peking Union Medical College, 167A Beilishi Road, Xi Cheng District, Beijing, 100037 China

Correction to: *Scientific Reports* 10.1038/srep20077, published online 29 January 2016

This Article contains errors. As a result of histology tissue sections in paraffin bath being mistakenly picked up during photographing, an incorrect histology panel was used in Figure 2.

The correct Figure 2 appears below as Figure 1.

**Figure 1:**
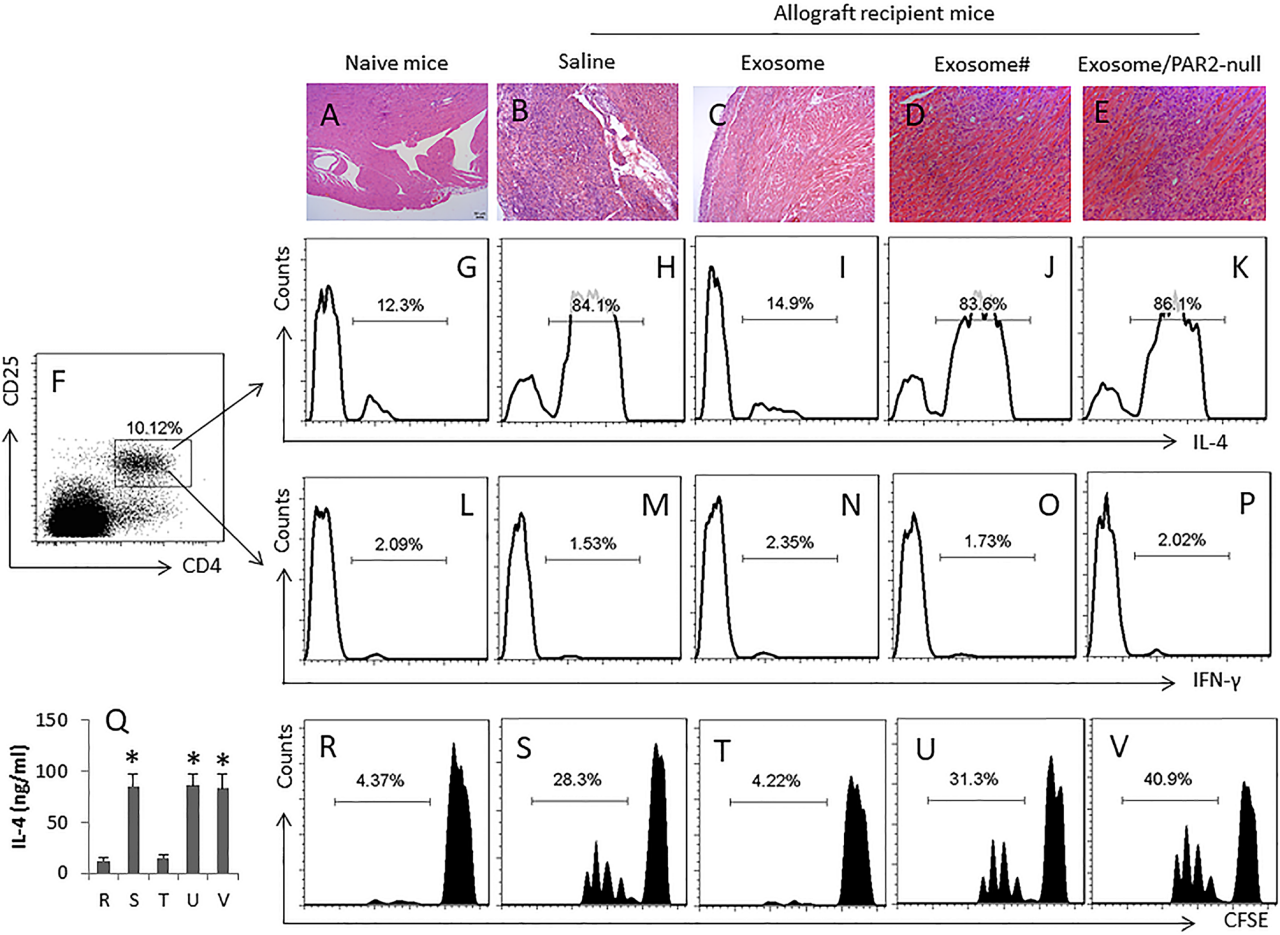
A correct version of the original Figure 2.

