# Supplementary material for: Donor-derived exosomes induce specific regulatory T cells to suppress immune inflammation in the allograft heart
Source: Sci Rep. 2016 Jan 29;7:20077. doi: 10.1038/srep20077 (PMC4731812; doi:10.1038/srep20077)
Supplement: Supplementary Information [file srep20077-s1.pdf]

Supplemental materials

**Donor-derived exosomes induce specific regulatory T cells to suppress immune inflammation in the allograft heart**

**Running title**: Peripheral exosomes suppress cardiac allograft inflammation

Jiangping Song*, Jie Huang*, Xiao Chen*, Xiao Teng, Zhizhao Song, Yong Xing, Mangyuan Wang, Kai Chen, Zheng Wang, Pingchang Yang, Shengshou Hu

State Key Laboratory of Cardiovascular Disease, Fuwai Hospital, National Center for Cardiovascular Diseases, Chinese Academy of Medical Sciences and Peking Union Medical College, 167A Beilishi Road, Xi Cheng District, Beijing, 100037, China.

*These authors contributed equally to this work.

**Corresponding authors**: Dr. Shengshou Hu; State Key Laboratory of Cardiovascular Disease, Fuwai Hospital, National Center for Cardiovascular Diseases, Chinese Academy of Medical Sciences and Peking Union Medical College, 167A Beilishi Road, Xi Cheng District, Beijing, 100037, China. Email: shrsrhu@163.com. Tel: 086-10-88396050; Fax: 086-10-88396052.

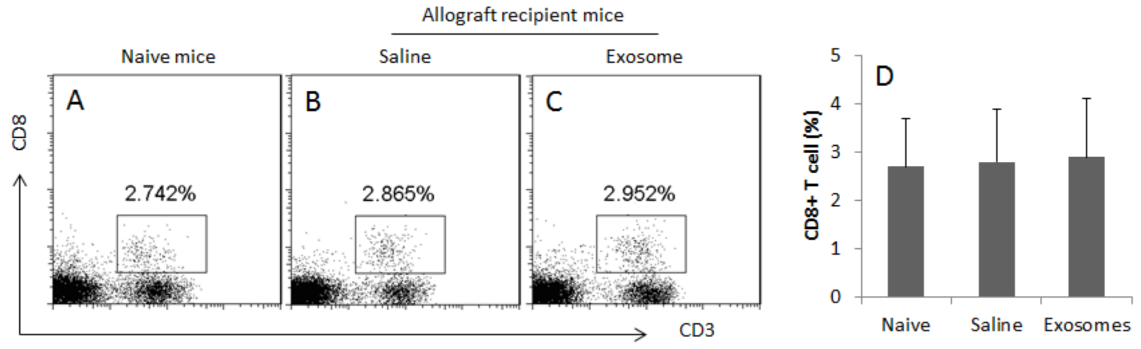


**FigS1. CD8$^+$ T cells in the heart**. Mononuclear cells were isolated from the heart of naive mice (A; n = 12) and allograft recipient mice (B, n = 12; C, n = 12). The cells were analyzed by flow cytometry. A-C, the gated plots indicate the frequency of CD3$^+$ CD8$^+$ T cells. D, the bars (mean ± SD) show the summarized data of A-C.
